# Recombinant salmonella-based 4-1BBL vaccine enhances T cell immunity and inhibits the development of colorectal cancer in rats: in vivo effects of vaccine containing 4-1BBL

**DOI:** 10.1186/1423-0127-20-8

**Published:** 2013-02-17

**Authors:** Jianxin Ye, Ling Li, Yuanting Zhang, Xueguang Zhang, Daming Ren, Weichang Chen

**Affiliations:** 1 Department of Gastroenterology, the First Affiliated Hospital of Soochow University, Suzhou 215006, China; 2 Institute of Medical Biotechnology, Key Laboratory of Medicine and Clinical Immunology of Jiangsu Province, Soochow University, 215007, Suzhou, China; 3 Institute of Genetics, State Key Laboratory of Genetic Engineering of Fudan University, 200433, Shanghai, China

**Keywords:** Recombinant attenuated salmonella, Colorectal cancer, 4-1BBL, T cell-based cellular immunity

## Abstract

**Background:**

Immunotherapy with vaccines is attractive for the treatment of cancer. This study is aimed at determining the effect of recombinant Salmonella (SL3261)-based 4-1BB ligand (4-1BBL) vaccine on the development of colorectal cancers and the potential immune mechanisms in rats.

**Results:**

In comparison with that in the PBS group, similar levels of 4-1BBL expression, the frequency of T cells, IFN-γ responses, and comparable numbers of tumors were detected in the SL3261 and SL3261C groups of rats. In contrast, significantly fewer numbers of tumors, increased levels of 4-1BBL expression in the spleens and colorectal tissues, higher frequency of peripheral blood and splenic CD3^+^CD25^+^ T cells, and stronger splenic T cell IFN-γ responses were detected in the SL3261R group of rats.

**Conclusion:**

Our results indicated that vaccination with recombinant attenuated Salmonella harboring the 4-1BBL gene efficiently enhanced T cell immunity and inhibited the development of carcinogen-induced colorectal cancers in rats.

## Background

Immunotherapy represents an attractive strategy for the prevention of tumor recurrence and the control of cancer growth [1,2]. Immunotherapy can induce antigen-specific T cell responses that can inhibit the growth of tumors. Effective activation of naïve T cells depends on TCR engagement and efficient co-stimulation [3,4]. The 4-1BB ligand (L) is expressed on antigen-presenting cells (APC), and can interact with 4-1BB on activated T cells, co-stimulating T cell cytokine production and proliferation [5-8], and prolonging T cell survival [7,9]. Therefore, 4-1BB and 4-1BBL are important regulators of T cell immunity.

Previous studies have shown that vaccination with antigen peptide linked with 4-1BBL enhances dendritic cell activation and antigen uptake and promotes CD8^+^ T cell effector/memory responses [10,11]. Similarly, the administration of both 4-1BBL and a HPV-specific E7 peptide or survivin protein eliminates E7-expressing TC-1 cervical cancer or survivin-expressing 3LL lung tumors in vivo [10,11]. Zhang et al. [12] reported that vaccination with natural CD137 ligand, is more efficient and less toxic side effects in inducing colorectal tumor regression in mice, as compared with treatment with anti-4-1BB agonist antibodies. In addition, our previous study has found that vaccination with a recombinant Salmonella-based 4-1BBL vaccine efficiently enhances T cell immunity in rats [13]. However, whether and how vaccination with a recombinant Salmonella-based 4-1BBL vaccine inhibits the development of colorectal tumors have not been fully illustrated.

In this present study, we investigated the effects of vaccination with a recombinant Salmonella-based 4-1BBL vaccine on the development of 1, 2-dimethylhydrazine(DMH)-induced colorectal tumors in rats. Our results indicated that vaccination with a recombinant Salmonella-based 4-1BBL vaccine effectively transferred the 4-1BBL gene into APCs and up-regulated 4-1BBL expression in APCs. Moreover, the up-regulation of 4-1BBL expression in APCs induced vigorous T cell immunity and inhibited the development of colorectal cancer in rats.

## Methods

### Bacteria

Salmonella typhimurium LB5000 and the attenuated Salmonella enterica serova typhimurium SL3261 were generously provided by Professor Stocker from Stanford University, USA. Plasmid pIRES2-EGFP was purchased from BD Biosciences Clontech (Palo Alto, CA, USA). The pIRES2-EGFP-transformed SL3261C or pIRES2-EGFP-4-1BBL-transformed SL3261R were generated, as previously described [13].

### Animals

A total of 20 male Sprague Dawley (SD) rats at 6–8 weeks of age were obtained from the Center of Experimental Animal of Soochow University. Rats were housed in a specific-pathogen-free (SPF) facility in 12-h light/dark cycle at 50 ± 10% humidity and 21 ± 2°C with free access to water and food. The body weights of individual rats were measured weekly. The experimental protocols were approved by the Ethics Committee of the First Affiliated Hospital of Soochow University.

### Establishment of rat model of colorectal tumor

Individual animals were administered subcutaneously with 20 mg/kg 1, 2-dimethylhydrazine (DMH, Sigma, St. Louis, USA) weekly for 20 consecutive weeks. Fourteen weeks after the first DMH administration, the rats were randomized and gavaged with 2 ml PBS, PBS containing 2 × 10^9^ SL3261, the pIRES2-EGFP-transformed SL3261, or the pIRES2-EGFP-4-1BBL-transformed SL3261 every two weeks for three times as the control, SL3261, SL3261C, or SL3261R group, respectively. At 21 weeks post the first DMH injection, the rats were anesthetized and their blood samples were collected and sacrificed. Their spleens were dissected, and a portion of the spleen from each rat was used for immunofluorescent staining.

### Gross and histological examination

The entire colorectal (from the cecum to the anus) tissues of individual rats were removed, washed thoroughly with ice-cold saline, cut longitudinally, and laid flat on a board. The numbers of tumors were counted in a blinded manner. The disease stages of colorectal cancers were evaluated by Duke’s stage system [14] with minor modifications. Briefly, Stage A: tumors only in the mucosa; Stage B-C, tumors invading into muscularis propria, but without distant metastasis; and Stage D: tumor with distant metastasis.

### Flow cytometric analysis

Peripheral blood mononuclear cells (PBMC) were isolated either at 16 weeks or 21 weeks post the initial DMH administration and splenic monnocnulear cells were prepared at 21 weeks post the initial DMH administration by Ficoll-paque density gradient centrifugation. The frequency of different subsets of T cells was characterized by flow cytometry. Briefly, 1 × 10^5^ cells were stained with anti-CD3-PE, anti-CD3-FITC, anti-CD8-FITC, anti-CD4-FITC, and anti-CD25-PE antibodies (Ebioscience), and after washing, the cells were subjected to flow cytometry and analyzed by FlowJo software.

### Reverse transcription-polymerase chain reaction (RT-PCR)

The relative levels of GFP mRNA transcripts in splenocytes were determined by RT-PCR. Briefly, total RNA was extracted from individual splenic mononuclear cell samples and reversely transcribed into cDNA using a specific kit TakaRa (Dalian, China), according to the manufacturers’ instruction. The sequences of primers were forward 5^′^-CACAAGTTCAGCGTGTCCG-3^′^ and reverse 5^′^-CTCGATGCGGTTCACCAG-3^′^ for GFP; forward 5^′^-GAGGGAAATCGTGCGTGAC-3^′^ and reverse 5^′^-TAGAAGCATTTGCGGTGC-3^′^ for β-actin. PCR amplification was performed in duplicate at 94°C for 5 min and subjected to 33 cycles of 94°C for 30 s, 49°C for 30 s, and 72ºC for 35 s, followed by 72°C for 10 min.

### Immunofluorescence

The levels of 4-1BBL and GFP expression were detected by immunofluorescence, as previously described [13]. In brief, the frozen spleen and colorectal tissue sections were fixed in Acetone and stained with goat anti-4-1BBL antibodies (CD137L, Santa Cruz Biotech, Santa Cruz, USA) and rabbit anti-GFP antibodies, respectively. After being washed, the bound antibodies were detected with donkey anti-goat-CY3 and goat anti-rabbit-FITC antibodies (Beyotime Institute of Biotechnology, Haimen, Jiangsu, China). Subsequently, the sections were mounted and examined under a confocal microscope (Leica, Germany).

### Enzyme-linked immunosorbent assay (ELISA)

Splenic monnocnulear cells were prepared at 21 weeks post the initial DMH administration by Ficoll-paque density gradient centrifugation. Splenic mononuclear cells (2.5 × 10^5^ cells/ml) were stimulated in triplicate with 10 μg/ml of PHA-P (Sigma) in RPMI1640 (GIBCO) medium containing 10% FCS and 2 μg/ml of Ciprobay (HealthCare, AG) for 72 hours. The supernatants of cultured cells were harvested and the concentrations of IFN-γ in the supernatants were measured by ELISA using a rat IFN-γ detection kit, according to the manufacturers’ instruction (Jingmei Biotech, Shenzhen, China).

### Statistical analysis

Data were expressed as the means ± SD. The difference among different groups was assessed by analysis of variance (ANOVA) and the difference between two groups was assessed by Student’s *t*-test. A P value of < 0.05 was considered statistically significant.

## Results

### Expression of reporter gene GFP and 4-1BBL protein

**Figure 1:**
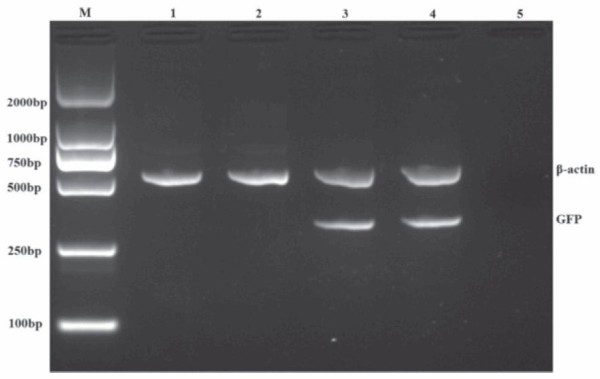
**RT-PCR analysis of GFP mRNA transcripts in splenocytes of the rats from different groups. **Following administration of DMH for 21 weeks, the rats were sacrificed and their splenocytes were isolated. Subsequently, the GFP mRNA transcripts in spelnocytes from different groups of rats were elevaluated by RT-PCR. β-actin was used as an internal control. Data shown are representative images from three separate experiments. Lane M, DNA marker; 1, PBS; 2, SL3261; 3, SL3261C; 4, SL3261R; 5, Negative control (RNA without reverse transcription).

**Figure 2:**
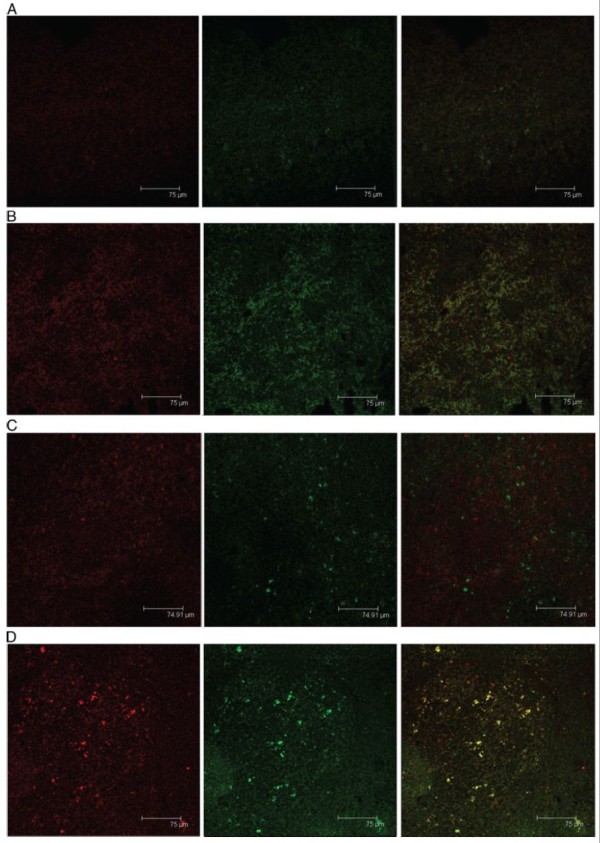
**Immunofuorescent analysis of 4-1BBL expression on rat spleens. **The expression of GFP and 4-1BBL in the spleens of different groups of rats was characterized by immunofuorescent assays using anti-4-1BBL (red) and anti-GFP (green) antibodies and examining under a confocal microscopy. Data shown are representative images of different groups of rats (n = 4 for the PBS group, n = 5 for other groups) from three separate experiments. (**A**) The PBS group; (**B**) The SL3261 group; (**C**) The SL3261C group; (**D**) The SL3261R group.

**Figure 3:**
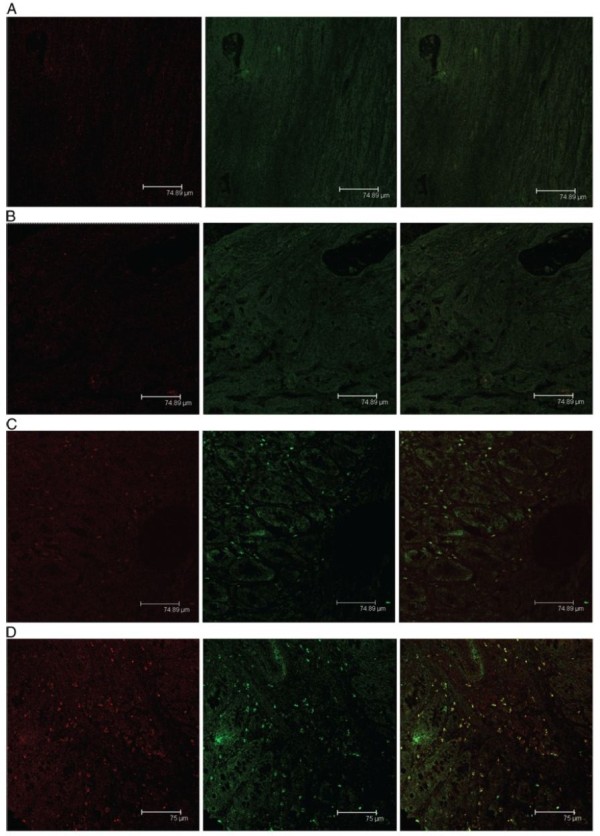
**Immunofuorescent analysis of 4-1BBL expression on the colorectal tumor tissues of rats. **The expression of GFP and 4-1BBL in the colorectal tumor tissues from different groups of rats was characterized by immunofuorescent assays using anti-4-1BBL (red) and anti-GFP (green) antibodies and examining under a confocal microscopy. Data shown are representative images of different groups of rats (n = 4 for the PBS group, n = 5 for other groups) from three separate experiments. (**A**) The PBS group; (**B**) The SL3261 group; (**C**) The SL3261C group; (**D**) The SL3261R group.

We first evaluated the reporter gene GFP mRNA transcription in splenocytes of different groups of rats using RT-PCR technique at 21-week post the initial DMH administration. We detected GFP mRNA transcripts in the spenocytes from the rats vaccinated with SL3261C or SL3261R (recombinant attenuated Salmonella typhimurium carrying pIRES2-EGFP or pIRES2-EGFP-4-1BBL plasmids), but not in the control and SL3261-vaccinated rats (Figure 1). Further immunofluorescent analysis revealed that GFP protein expression was detected in the spleen and colorectal tissues from the SL3261C- and SL3261R-vaccinated rats (Figures 2 and 3). In addition, analysis of the 4-1BBL expression revealed that the intensity of anti-4-1BBL staining increased in the spleen and colorectal tissues from the SL3261R-vaccinated rats. These observations indicated that vaccination with the SL3261R effectively enhanced 4-1BBL expression in these tissues of rats.

### Vaccination with the SL3261R reduces the numbers of colorectal tumors in rats

**Figure 4:**
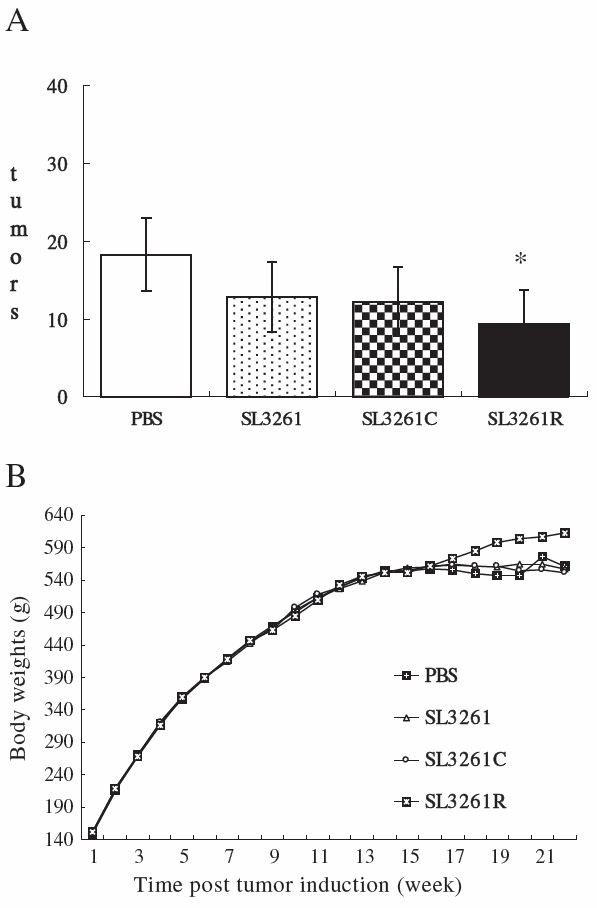
**The body weights and the number of tumors in different groups of rats. **Following induction of colorectal tumors, the body weights of individual rats were measured longitudinally and the numbers of colorectal tumors in individual rats at 21 weeks post the induction were examined in a blinded manner. Data are expressed as the mean or mean ± SD of different groups of rats (n = 5 per group). (**A**) The numbers of tumors. (**B**) The body weights of animals. *P < 0.05 SL3261R vs. PBS.

Following the induction of colorectal tumors, one out of 20 rats died at 20 weeks post the initial DMH treatment. Characterization of animals indicated that a large amount of bloody ascites was found in the rats. A great number of tumor nodules varying in size were observed in the mesentery and posterior peritoneum. The rigor of the colorectal rectal wall appeared. A total of 18 adenocarcinoma were identified by histological examination using H & E staining and visualized in the entire colorectal of the dead rats (data not shown). In addition, the numbers of tumors at 21 weeks post DMH treatment were 18.2 ± 4.7 in the PBS group; 12.8 ± 4.5 in the SL3261-treated group; 12.2 ± 4.4 in the SL3261C-treated group; and 9.4 ± 4.3 in the SL3261R-treated group (Figure 4A). The number of tumors in the SL3261R-treated group of rats was significantly less than that in the PBS group. In addition, we found that most of the tumors in the PBS group of rats were at Dukes’ stage D (stage B-C, 1; Stage D, 4), while most tumors in the SL3261 and SL3261C groups were at stage B-C (SL3261: stage A, 1; stage B-C, 2; Stage D, 2; SL3261C: stage B-C, 4; stage D, 1). However, the tumors in the SL3261R group of rats were at stage A-C (Stage A, 2; Stage B-C, 2) (Table 1). Moreover, there was no significant difference in the rat body weights among the four groups, although slightly increased body weights were observed in the SL3261R-vaccinated group of rats 16 weeks after DMH application (Figure 4B).

**Table 1:** Analysis of tumor staging in rats from different experimental groups

**Groups**	**Negative**	**Stage A**	**Stage B-C**	**Stage D**
PBS	0	0	1	4
SL3261	0	1	2	2
SL3261C	0	0	4	1
SL3261R	1	2	2	0

The number of animals in Dukes Stage A, Stage B-C or Stage D was listed.

### T cell phenotypes

**Figure 5:**
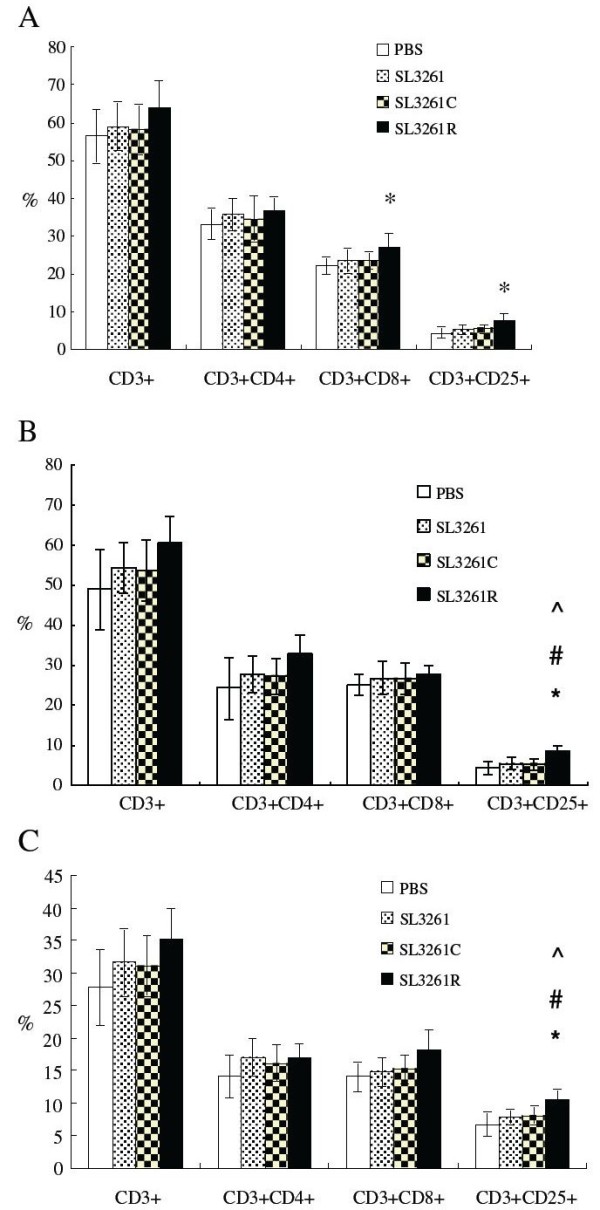
**The frequency of different subsets of T cells. **Following induction of colorectal tumors for 16 weeks, peripheral blood samples were obtained from individual rats and the frequency of CD3+, CD3 + CD4+, CD3 + CD8+ and CD3 + CD25+ T cells was characterized by flow cytometry analysis (**A**). At 21 weeks post induction, the rats were sacrificed and their peripheral blood (**B**) and splenic T cells (**C**) were characterized by flow cytometry analysis. Data are expressed as the mean ± SD of different groups of rats (n = 4 for the PBS group, n = 5 for other groups) from three separate experiments. *P < 0.05 SL3261R vs. PBS; #*P* < 0.05 SL3261R vs. SL3261; ^*P* < 0.05 SL3261R vs. SL3261C.

To understand the anti-tumor effect, the frequency of different subsets of peripheral and splenic T cells was characterized by flow cytometry analysis at 16 weeks post the initial DMH administration. We found that the frequency of peripheral blood CD3^+^CD8^+^ and CD3^+^CD25^+^ T cells, but not the total CD3^+^ and CD3^+^CD4^+^ T cells, in the SL3261R group was significantly higher than that in the PBS control (P < 0.05) (Figure 5A). No significant difference was found in the number of CD3^+^, CD3^+^CD4^+^ and CD3^+^CD8^+^ T cells of peripheral blood and splenic T cells among PBS control, SL3261 and SL3261C groups 21 weeks after the initial DMH administration (P > 0.05). But CD3^+^CD25^+^ T cells in the SL3261R group was significantly higher than that in the other groups (P < 0.05) (Figure 5B-C).

### Vaccination with the SL3261R enhances splenic IFN-γ responses

**Figure 6:**
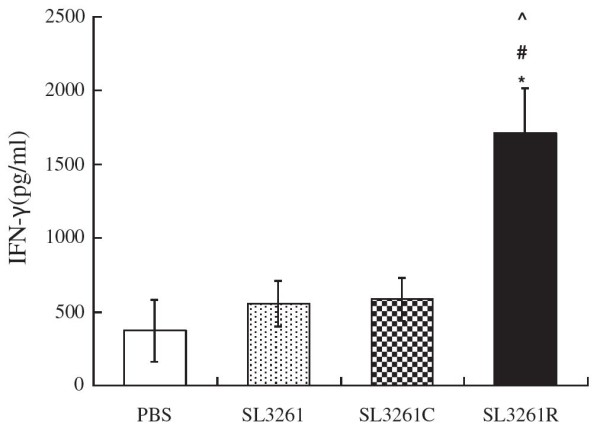
**ELISA analysis of the concentrations of IFN-γ in the supernatants of cultured splenocytes. **Splenic mononuclear cells were isolated from individual rats at 21 weeks post induction and stimulated with PHA for 72 h. The concentrations of IFN-γ in the supernatants of cultured splenocytes were measured by ELISA. Data are expressed as the mean ± SD of different groups of rats (n = 4 for the PBS group, n = 5 for other groups) from three separate experiments. *P < 0.01 SL3261R vs. PBS; #*P* < 0.01 SL3261R vs. SL3261; ^*P* < 0.01 SL3261R vs. SL3261C.

The concentrations of IFN-γ in the cultured supernatants of splenocytes stimulated with PHA from individual groups of rats were analyzed by ELISA. As shown in Figure 6, the levels of IFN-γ were comparable among the PBS, SL3261, and SL3261C-treated rats. In contrast, the concentrations of IFN-γ in the cultured splenocytes from the SL3261R-vaccinated rats were significantly higher than that in the other groups of rats (P < 0.01). However, no significant difference was detected among PBS, SL3261, and SL3261C groups (P > 0.05).

## Discussion

The 4-1BBL is a type II surface glycoprotein of the tumor necrosis factor (TNF) superfamily, and is generally expressed in APCs, such as dendritic cells, macrophages, and activated B cells [4]. 4-1BBL binds to 4-1BB (also called CD137), a member of the TNFR superfamily, and enhances T cell activation [4]. Emerging lines of evidence shows that activation of 4-1BB by agonistic monoclonal antibodies (Abs) or over-expressing 4-1BBL enhances T cell proliferation and cytokine production, up-regulates anti-apoptotic gene expression and prevents activation-induced cell death [15-17]. However, systemic administration of anti-4-1BB agonistic Abs is unsuccessful in correcting the defect in response to severe influenza in 4-1BBL-deficient mice, which is possibly because anti-4-1BB can target many cell types and lead to high levels of cytokine production and immunopathology [18,19]. A HIV-1 effective vaccine should induce both antigen-specific cellular and humoral immunity and DNA vaccine for inducing 4-1BBL expression is a superior adjuvant to enhance HIV-specific immunity than anti-4-1BB agonistic antibody [20]. Based on these findings, our previous study has shown that recombinant attenuated Salmonella harboring the 4-1BBL gene can induces high levels of 4-1BBL expression in dendritic and other immune cells [13]. In the current study, we explored the effect of oral treatment with a recombinant Salmonella-based 4-1BBL vaccine on the development of DMH-induced colorectal cancers in rats and the potential immune mechanisms.

A previous study indicated that abnormal morphology characterized by accumulation of undifferentiated cells with pleiomorphic and conspicuous nucleoli in a small cluster of neighboring crypts was found at 12 weeks post DMH administration. Microscopic carcinomatous foci were observed at 15 weeks post DMH treatment [21]. Therefore, in this present study, the vaccine administration was started 14 weeks after the DMH treatment.

Inoculation of recombinant Salmonella appeared to induce more 4-1BBL expression in the spleens and colorectal tumor tissues. Evidentially, we detected the reporter GFP mRNA transcripts and protein expression only in the spleens from the SL3261C and SL3261R groups of rats. Similarly, there was more 4-1BBL expression in the spleens and colorectal tissues than the other groups. In this study, we found that SL3261R suppressed tumor growth as compared with PBS group (P < 0.05). However, no significant difference was found between SL3261R group and SL3261 or SL3261C group (P > 0.05), although slightly decreased number of colorectal tumors was observed in SL3261R group. As Salmonella can localize to transplantable murine tumors and partially inhibit tumor growth [22], it is possible that the attenuated Salmonella (SL3261) itself may have anti-tumor effect. We further staged the colon cancer and found that most of the tumors in PBS groups were in late stage (Dukes’ stage D). In SL3261 and SL3261C groups, tumors were found in middle and late stages (Dukes’ stage C D), whereas tumors in SL3261R group were in early and middle stages (Dukes’ stage A B C). Together, our data extended previous findings and indicated that recombinant bacteria-based 4-1BBL vaccines effectively inhibited the development of colorectal cancer [12].

CD3^+^CD8^+^ cells are major players of T cell immunity against tumors [23]. CD25 is the α-chain of IL-2R, which is absent on naive resting T cells, except for T regulatory cells. Following stimulation by “signal two”, activated T cells initiate IL-2 expression and secretion, which acts as an autocrine growth factor because activated T cells express high-affinity IL-2R. IL-2 exerts its pleiotropic activities through binding to either dimeric receptor composed of β-chain IL-2R (CD122) and common cytokine receptor γ-chain (CD132) or the receptor composed of trimeric IL-2R. alpha; IL-2Rβ, and common cytokine receptor γ-chain. CD25 has been considered as a marker of activated lymphocytes [24,25]. Consistent with our previous results [13], the number of CD3 + CD8+ and CD3 + CD25+ T cells in peripheral blood obtained from SL3261R group was increased following induction of colorectal tumors for 16 weeks, when animals were given drug administration for once (P < 0.05 compared with PBS group). Nevertheless, no statistical difference was found between SL3261R group and SL3261 or SL3261C group (P > 0.05), which might be due to the short time period of drug administration and only once vaccine administration. Significant elevated number of CD3 + CD25+ T cells in peripheral blood was detected 21 weeks following tumor induction, when animals were given 3 times of drug administration in SL3261R group (P < 0.05 compared with all groups), suggesting the recombinant bacteria-based 4-1BBL vaccines can enhance the immune function of tumor-bearing mice. We did not observe significant difference in the frequency of peripheral blood and splenic CD3^+^CD8^+^ T cells between the SL3261R and other groups of rats at 21 weeks post DMH treatment, It is possible that CD3^+^CD8^+^ T cells may migrate into the tumors with the progression of tumorigenesis. More importantly, we detected significantly higher levels of splenic IFN-γ responses in the SL3261R group of rats. Given that IFN-γ is predominantly secreted by Th1 and cytotoxic CD8^+^ T cells (the stronger IFN-γ responses), together with a higher frequency of activated T cells suggest that the recombinant vaccine may enhance T cell immunity that inhibits the development of colorectal cancers in rats [26].

## Conclusions

In summary, our data indicated that recombinant Salmonella-based 4-1BBL vaccine induced 4-1BBL expression in immune cells and administration of the vaccine efficiently inhibited the development of DMH-induced colorectal tumors in rats, accompanied by increasing the frequency of activated T cells and splenic IFN-γ responses. Our findings may provide a new basis for the design of immunotherapies for the intervention of colorectal cancer.

## Competing interests

The authors declare that they have no competing interests.

## Authors’ contributions

JXY and YTZ performed research; JXY and LL wrote the manuscript and analyzed data; DMR and XGZ performed the partial experiments; JXY and WCC conceived of the study and designed research. All authors read and approved the final manuscript.

## Highlights

Recombinant Salmonella-based 4-1BBL vaccine effectively enhanced 4-1BBL expression.

This Vaccine inhibited the development of colorectal cancer in rats.

Vaccine enhanced T cell immunity in rats.

## References

[B1] Rosenberg SA Yang JC Restifo NP Cancer immunotherapy: moving beyond current vaccines Nat Med 2004 10 909 915 10.1038/nm1100 15340416 PMC1435696

[B2] Vinay DS Kwon BS Immunotherapy of cancer with 4-1BB Mol Cancer Ther 2012 11 1062 1070 10.1158/1535-7163.MCT-11-0677 22532596

[B3] Hodge JW Greiner JW Tsang KY Sabzevari H Kudo-Saito C Grosenbach DW Gulley JL Arlen PM Marshall JL Panicali D Schlom J Costimulatory molecules as adjuvants for immunotherapy Front Biosci 2006 11 788 803 10.2741/1837 16146771

[B4] Cheuk AT Mufti GJ Guinn BA Role of 4-1BB: 4-1BB ligand in cancer immunotherapy Cancer Gene Ther 2004 11 215 226 10.1038/sj.cgt.7700670 14671675

[B5] Laderach D Movassagh M Johnson A Mittler RS Galy A 4-1BB co-stimulation enhances human CD8(+) T cell priming by augmenting the proliferation and survival of effector CD8(+) T cells Int Immunol 2002 14 1155 1167 10.1093/intimm/dxf080 12356681

[B6] Shuford WW Klussman K Tritchler DD Loo DT Chalupny J Siadak AW Brown TJ Emswiler J Raecho H Larsen CP 4-1BB costimulatory signals preferentially induce CD8+ T cell proliferation and lead to the amplification in vivo of cytotoxic T cell responses J Exp Med 1997 186 47 55 10.1084/jem.186.1.47 9206996 PMC2198949

[B7] Cannons JL Lau P Ghumman B DeBenedette MA Yagita H Okumura K Watts TH 4-1BB ligand induces cell division, sustains survival, and enhances effector function of CD4 and CD8 T cells with similar efficacy J Immunol 2001 167 1313 1324 11466348 10.4049/jimmunol.167.3.1313

[B8] Cooper D Bansal-Pakala P Croft M 4-1BB (CD137) controls the clonal expansion and survival of CD8 T cells in vivo but does not contribute to the development of cytotoxicity Eur J Immunol 2002 32 521 529 10.1002/1521-4141(200202)32:2<521::AID-IMMU521>3.0.CO;2-X 11828369

[B9] Lee HW Park SJ Choi BK Kim HH Nam KO Kwon BS 4-1BB promotes the survival of CD8+ T lymphocytes by increasing expression of Bcl-xL and Bfl-1 J Immunol 2002 169 4882 4888 12391199 10.4049/jimmunol.169.9.4882

[B10] Sharma RK Elpek KG Yolcu ES Schabowsky RH Zhao H Bandura-Morgan L Shirwan H Costimulation as a platform for the development of vaccines: a peptide-based vaccine containing a novel form of 4-1BB ligand eradicates established tumors Cancer Res 2009 69 4319 4326 10.1158/0008-5472.CAN-08-3141 19435920 PMC2755220

[B11] Sharma RK Schabowsky RH Srivastava AK Elpek KG Madireddi S Zhao H Zhong Z Miller RW Macleod KJ Yolcu ES Shirwan H 4-1BB ligand as an effective multifunctional immunomodulator and antigen delivery vehicle for the development of therapeutic cancer vaccines Cancer Res 2010 70 3945 3954 10.1158/0008-5472.CAN-09-4480 20406989 PMC2872136

[B12] Zhang N Sadun RE Arias RS Flanagan ML Sachsman SM Nien YC Khawli LA Hu P Epstein AL Targeted and untargeted CD137L fusion proteins for the immunotherapy of experimental solid tumors Clin Cancer Res 2007 13 2758 2767 10.1158/1078-0432.CCR-06-2343 17460060

[B13] Ye JX Zhang YT Zhang XG Ren DM Chen WC Recombinant attenuated Salmonella harboring 4-1BB ligand gene enhances cellular immunity Vaccine 2009 27 1717 1723 10.1016/j.vaccine.2009.01.031 19187795

[B14] Kyriakos M The President’s cancer, the Dukes classification, and confusion Arch Pathol Lab Med 1985 109 1063 1066 3907586

[B15] Watts TH TNF/TNFR family members in costimulation of T cell responses Annu Rev Immunol 2005 23 23 68 10.1146/annurev.immunol.23.021704.115839 15771565

[B16] Croft M Co-stimulatory members of the TNFR family: keys to effective T-cell immunity? Nat Rev Immunol 2003 3 609 620 10.1038/nri1148 12974476

[B17] Lim HY Kim KK Zhou FC Yoon JW Hill JM Kwon BS 4-1BB-like molecule is expressed in islet-infiltrating mononuclear cells and in the gray matter of the brain Cell Biol Int 2002 26 271 278 10.1006/cbir.2001.0847 11991655

[B18] Niu L Strahotin S Hewes B Zhang B Zhang Y Archer D Spencer T Dillehay D Kwon B Chen L Cytokine-mediated disruption of lymphocyte trafficking, hemopoiesis, and induction of lymphopenia, anemia, and thrombocytopenia in anti-CD137-treated mice J Immunol 2007 178 4194 4213 17371976 10.4049/jimmunol.178.7.4194 PMC2770095

[B19] Lin GH Sedgmen BJ Moraes TJ Snell LM Topham DJ Watts TH Endogenous 4-1BB ligand plays a critical role in protection from influenza-induced disease J Immunol 2009 182 934 947 19124736 10.4049/jimmunol.182.2.934

[B20] Ganguly S Liu J Pillai VB Mittler RS Amara RR Adjuvantive effects of anti-4-1BB agonist Ab and 4-1BBL DNA for a HIV-1 Gag DNA vaccine: different effects on cellular and humoral immunity Vaccine 2010 28 1300 1309 10.1016/j.vaccine.2009.11.020 19944789 PMC2814905

[B21] Maskens AP Histogenesis and growth pattern of 1,2-dimethylhydrazine-induced rat colon adenocarcinoma Cancer Res 1976 36 1585 1592 178424

[B22] Rosenberg SA Spiess PJ Kleiner DE Antitumor effects in mice of the intravenous injection of attenuated Salmonella typhimurium J Immunother 2002 25 218 225 10.1097/00002371-200205000-00004 12000863 PMC2413432

[B23] Schiavon V Roth P Bolton WE Farcet JP Bensussan A Boumsell L Lymphocytes subsets in normal individuals: analysis by four color immunofluorescence and flow cytometry on whole blood Tissue Antigens 1996 48 312 318 10.1111/j.1399-0039.1996.tb02650.x 8946685

[B24] Stauber DJ Debler EW Horton PA Smith KA Wilson IA Crystal structure of the IL-2 signaling complex: paradigm for a heterotrimeric cytokine receptor Proc Natl Acad Sci USA 2006 103 2788 2793 10.1073/pnas.0511161103 16477002 PMC1413841

[B25] Tomala J Chmelova H Mrkvan T Rihova B Kovar M In vivo expansion of activated naive CD8+ T cells and NK cells driven by complexes of IL-2 and anti-IL-2 monoclonal antibody as novel approach of cancer immunotherapy J Immunol 2009 183 4904 4912 10.4049/jimmunol.0900284 19801515

[B26] Reyes E Prieto A de la Hera A de Lucas P Alvarez-Sala R Alvarez-Sala JL Alvarez-Mon M Treatment with AM3 restores defective T-cell function in COPD patients Chest 2006 129 527 535 10.1378/chest.129.3.527 16537848

